# Low-Dose Oral Ginger Improves Daily Symptom Scores in Asthma

**DOI:** 10.3390/ph17121651

**Published:** 2024-12-08

**Authors:** Charles W. Emala, Tarnjot K. Saroya, Yuqi Miao, Shuang Wang, Shengmin Sang, Emily A. DiMango

**Affiliations:** 1 Department of Anesthesiology, Columbia University Vagelos College of Physicians and Surgeons, 628 W. 168th St. PH 505 Center, New York, NY 10032, USA; 2 Department of Medicine (Pulmonology, Allergy and Critical Care), Columbia University Vagelos College of Physicians and Surgeons, New York, NY 10032, USA; 3 Department of Biostatistics, Mailman School of Public Health, Columbia University, New York, NY 10027, USA; 4 Laboratory for Functional Foods and Human Health, Center for Excellence in Post-Harvest Technologies, North Carolina Agricultural and Technical State University, North Carolina Research Campus, Kannapolis, NC 28081, USA

**Keywords:** methacholine, exhaled nitric oxide, cytokines, eosinophils, asthma control test, AQLQ, 2-week symptom recall

## Abstract

**Background/Objective:** A significant number of individuals with asthma have poorly controlled daily symptoms and utilize dietary supplements such as ginger in a quest for improved symptom control; however, its effectiveness at improving the control of symptoms is unproven. We questioned whether low-dose oral ginger would improve subjective and objective measurements of asthma control in mild-to-moderate asthmatics. **Methods:** We performed a randomized, placebo-controlled, double-blinded study of a low dose (1 g twice daily) of a dietary supplement of ginger in 32 mild-to-moderate uncontrolled asthmatics over a 2-month trial period while maintaining daily conventional asthma therapies. The planned primary outcomes included an increased tolerance to inhaled methacholine and decreased concentrations of fractional excretion of exhaled nitric oxide (FeNO). Secondary planned outcomes included measurements of asthma control by the Asthma Control Test (ACT), a 2-week symptom recall test, and the Juniper mini Asthma Quality of Life Questionnaire (AQLQ), and blood eosinophils and asthma-associated cytokines. **Results:** Exhaled nitric oxide or blood eosinophils were not changed by oral ginger. However, three different measures of asthma symptom control were improved by the 28-day time point of oral ginger. Asthma-associated serum cytokines (IL-13 and IL-17A) were modulated by oral ginger. **Conclusions:** This is the first demonstration that a small daily dose of a dietary supplement of ginger may improve asthma symptoms and reduce inflammation in human asthmatics. These findings support the need for additional studies using larger doses of ginger in specific endotypes of asthmatics that may identify a novel therapeutic for asthma.

## 1. Introduction

Asthma is a chronic inflammatory disorder of the airways characterized by bronchoconstriction and airway inflammation. Presently, an estimated 25.3 million Americans suffer from asthma, and over 10 million have at least one asthma attack per year [1]. Current asthma therapies include short- and long-acting β-agonists to combat bronchoconstriction and inhaled corticosteroids to reduce airway inflammation. These conventional therapies inadequately control symptoms in 40% of asthmatics [2,3]. Targeted biologic therapies are aimed at patients with severe asthma, are expensive, and do not address the multiple endotypes of asthma [4,5,6,7].

The use of naturally derived therapeutics for asthma began with the use of methylxanthines, including caffeine, in the early 20th century. Current research efforts directed at complementary and alternative (CAM) therapies for asthma by others have demonstrated that an Anti-Asthma Herbal Medicine Intervention (ASHMI) reduces lung inflammation, airway remodeling, and airway smooth muscle hyperresponsiveness [8,9,10,11,12]. These studies support efforts to identify novel bronchodilators and anti-inflammatory agents derived from natural sources.

Recent studies found that 60% of moderate asthmatics and 70% of severe asthmatics report using CAM to self-treat their asthma symptoms [13]. Of the many types of CAM therapies chosen by asthmatics, as many as 40% of asthmatics use herbal therapies to self-treat their asthma symptoms [14,15]. The exact mechanism of action of these agents is unclear but may involve attenuation of the allergic cascade through blockade of IgE-mediated inflammation [16], anti-inflammatory and antioxidant effects [17], and/or direct effects on airway smooth muscle (ASM) [11,18].

Ginger and its active constituents have been shown to reduce airway cell inflammation [19] and airway remodeling in a mouse model [20]. We have previously reported the bronchorelaxant properties of ginger in ex vivo airway smooth muscle, with evidence of 6-gingerol, 8-gingerol, and 6-shogaol as the active components inducing bronchorelaxation. In addition, inhalation of 8-gingerol protected against methacholine-induced airway hyper-responsiveness in an in vivo mouse model of asthma [21]. Cell-signaling pathways mediating contraction and relaxation of ASM are well characterized. A key enzyme that controls contractile tone in ASM is Gq-activated phospholipase Cβ. Our published study demonstrates that ginger components can inhibit this enzyme, which would yield a potent pro-relaxant effect via both reduced intracellular Ca^2+^ levels and reduced Ca^2+^ sensitivity of contractile phosphoproteins (i.e., MLC_20_) [22]. Clinical trials with chronic oral ginger therapy have demonstrated safe consumption of 2 g/day and resulted in beneficial effects in human colon cancer [23,24] and other inflammatory diseases in vivo [25]. Smooth muscle of the gut and airway share many signaling pathways, suggesting that ginger and its constituents may have direct effects on ASM and may contribute to bronchodilation. While pre-clinical studies establish that ginger and its active biologic components relax isolated human airway smooth muscle [21,22] and reduce airway inflammation in a mouse model of asthma [26], it is unknown if this translates into a clinical benefit in human asthmatics. Multiple prior studies in rodent models of asthma [26,27,28] and cell-based models of inflammation [29] have demonstrated a benefit of ginger or ginger constituents in reducing lung inflammation and airway hyperresponsiveness; however, no controlled clinical studies in human asthmatics have evaluated the potential benefits of ginger. Therefore, we conducted a placebo-controlled, double-blinded pilot study of a low daily dose of a dietary supplement of ginger extract in mild-to-moderate asthmatics to identify a potential benefit on asthma symptoms and inflammation.

## 2. Results

Study subjects were enrolled from September 2019 through February 2021, with a three-month research pause from March through May 2020 due to the COVID-19 pandemic. Due to institutional suspension of aerosol-generating methacholine challenge testing immediately following the COVID-19 pause, an insufficient number of study subjects completed methacholine challenge testing to allow for analysis.

**Figure 1:**
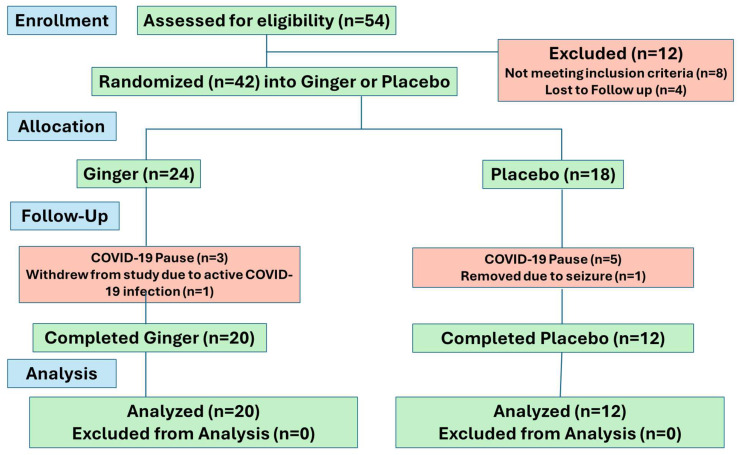
Consort flow diagram of subjects screened and randomized.

Fifty-four participants were screened. There were 12 screen failures (4 lost to follow-up and 8 did not meet inclusion criteria because 2 subjects had well-controlled asthma, 2 subjects had an FEV1 < 60%, 3 subjects were on exclusionary drugs, and 1 subject was not taking any asthma medications). Moreover, 42 participants were randomly assigned to either the ginger or placebo group. Eight subjects were active in this study at the start of the COVID-19 pause and were discontinued (three in the ginger group and five in the placebo group); their data are not included in the analysis. An additional subject in the placebo arm was withdrawn by the investigator due to seizure, and one participant in the ginger group withdrew due to an active COVID-19 infection. These subjects did not complete study visits and are not included in the analysis. Furthermore, 32 participants completed this study, i.e., 20 in the ginger group and 12 in the placebo group (please see the CONSORT diagram in Figure 1).

The baseline characteristics of participants who completed this study are summarized in Table 1.

There was no significant difference in baseline characteristics between groups. There was one serious adverse event of hospitalization due to viral illness in a subject randomized to placebo. This participant completed the study. Compliance in the planned consumption of the study drug in the ginger group at visit 4 and visit 5 was 91 ± 3.1% and 90 ± 2.9%, respectively (mean ± SEM), and 80 ± 5.8% and 83 ± 6.0%, respectively, in the placebo group (*p* = 0.08 between ginger and placebo at visit 4 and *p* = 0.24 between ginger and placebo at visit 5).

The initial primary outcome, methacholine PD20, was only successfully measured across the intended study visits (visits 2, 4, and 5) in 15 subjects (11 in ginger, 4 in placebo) due to discontinuation of methacholine bronchoprovocation after the COVID-19 research pause. Spirometry values were compared between the ginger and placebo groups at the randomization visit (visit 3) using two-sample t-tests, and there is no significant difference between the two groups. Table 2 shows the change in spirometry values between baseline and 1 h after ingestion of the first dose of the study drug at randomization (V3), within the ginger group and within the placebo group, separately, to examine if there are short-term effects on spirometry. No significant short-term effects in spirometry were detected in either group. One hour following ingestion of the study drug, there was no significant difference in any spirometric measure in either the ginger or placebo group (mean change of −2.42% in FEV1 in the ginger group (*p* = 0.58) and −3.72% in the placebo group (*p* = 0.52) (Table 2).

In addition to comparing changes in spirometry values *within* the ginger and placebo groups, we also compared changes in spirometric values *between* the two groups and found no statistically significant differences. Table 3 shows the regression coefficient estimates of the group indicator (ginger vs. placebo) and their 95% CIs from a linear mixed model examining if the overall long-term effects on spirometric measures (V3–V5) are different between the ginger and placebo groups. No significant differences in these spirometric measures were detected.

**Figure 2:**
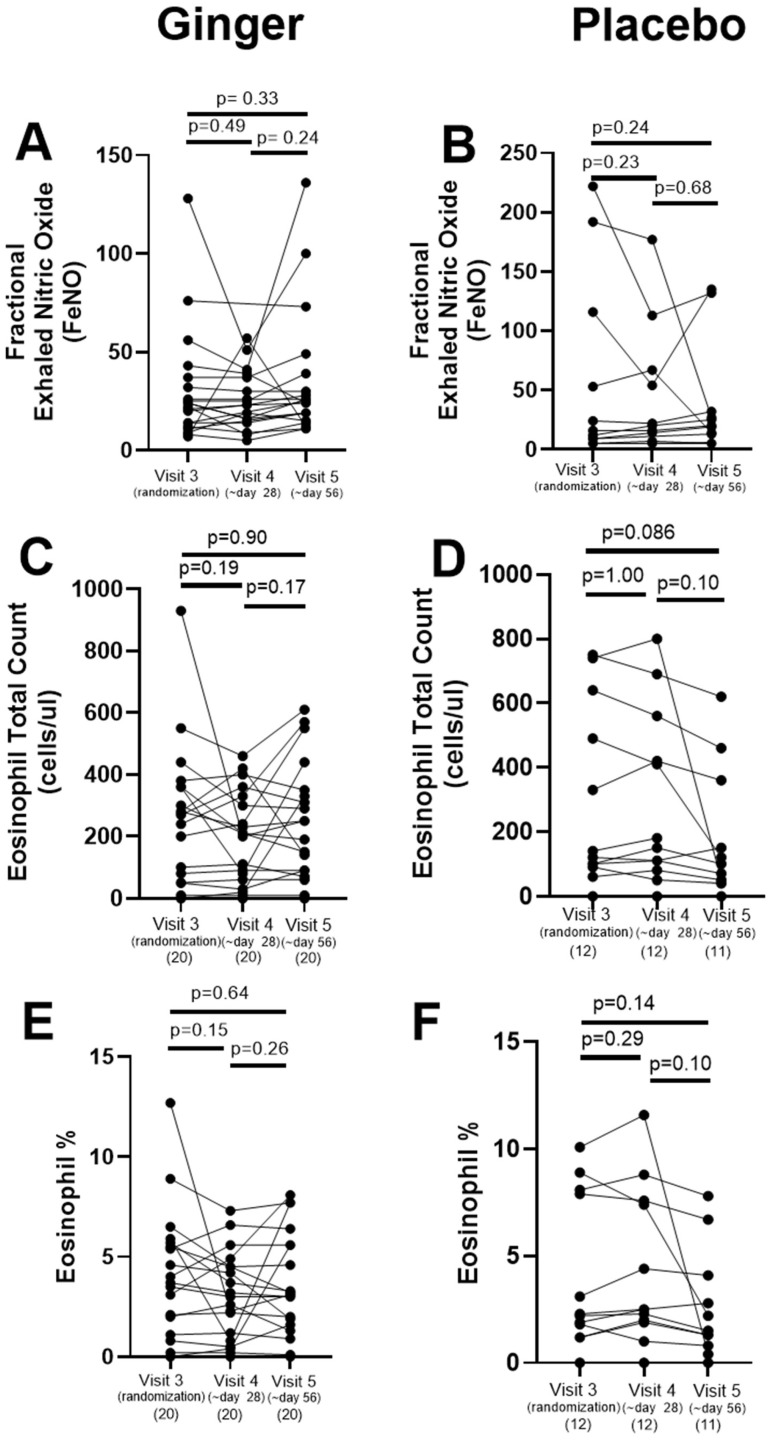
Exhaled fraction of nitric oxide (FeNO) and blood eosinophils were measured at the randomization visit (visit 3) and after 28 days (visit 4) or 56 days (visit 5) of study drug. (**A**,**B**) There were no statistically significant changes in FeNO across the study visits in either the ginger (n = 20) or placebo (n = 12) groups. There were no statistically significant changes in (**C**,**D**) total or (**E**,**F**) the percentages of blood eosinophils across the study visits in either the ginger (n = 20) or placebo (n = 11–12) groups.

FeNO or blood eosinophils did not differ between visits within the ginger group or within the placebo group (Figure 2). Data points were missing for one subject in the placebo group for the percent of eosinophils at visit 5 due to an inadequate blood draw.

**Figure 3:**
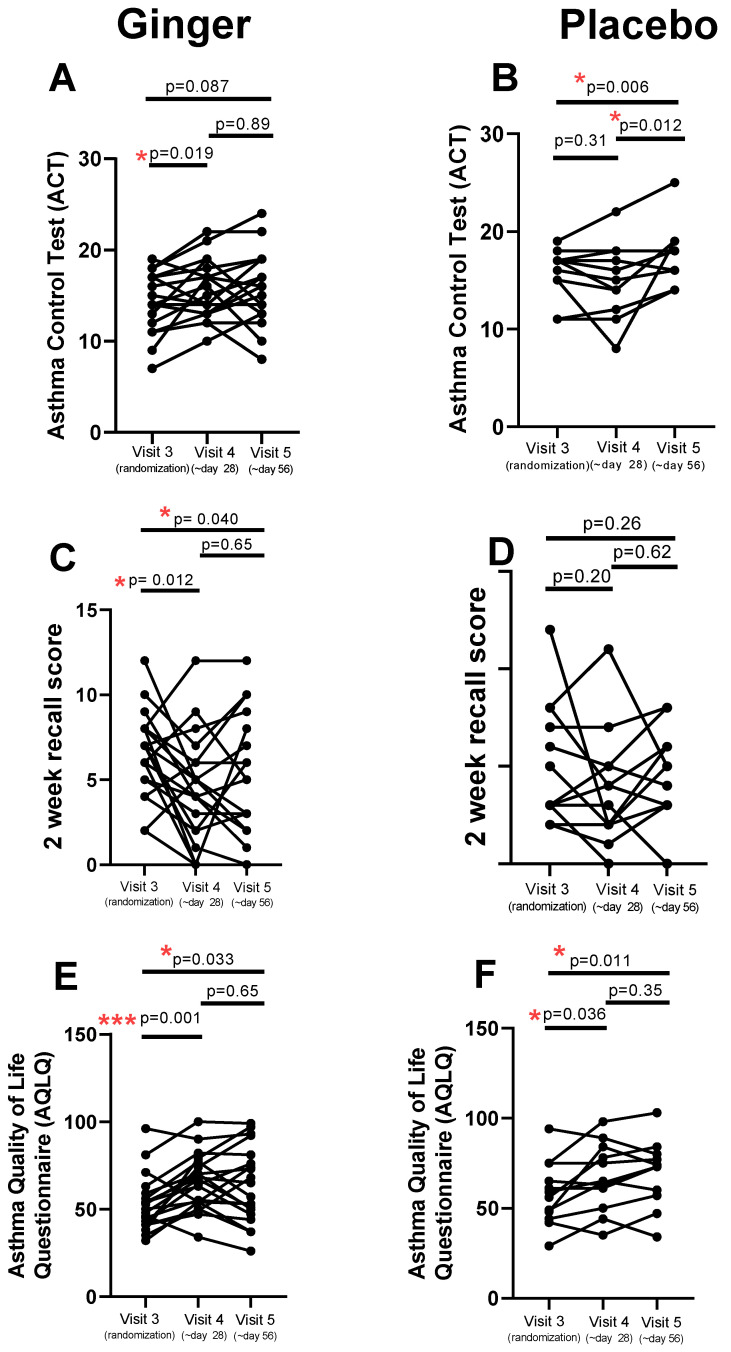
Asthma symptom scores were recorded by three methods (Asthma Control Test (ACT), 2-week recall score, and mini Asthma Quality of Life Questionnaire (AQLQ)) at the randomization visit (visit 3) and after 28 days (visit 4) or 56 days (visit 5) in subjects randomized to ginger (n = 20) or placebo (n = 12). (**A**) Ginger significantly improved scores on the asthma control test (ACT) between visits 4 and 5 (* *p* = 0.019). (**B**) Placebo also significantly reduced ACT scores between visits 3 to 5 (* *p* = 0.006) and between visits 4 to 5 (* *p* = 0.012). (**C**) Ginger improved the two week asthma symptom recall score between visits 3 to 4 (* *p* = 0.012) and between visits 3 to 5 (* *p* = 0.040). (**D**) Placebo had no effect on 2 week asthma symptom recall scores. (**E**) Ginger improve Asthma Quality of Life scores between visits 3 to 4 (* *p* = 0.033) and between visits 3 to 5 (*** *p* = 0.001). (**F**) Placebo had smaller effects on the Asthma Quality of Life scores between visits 3 to 4 (* *p* = 0.036) and visits 3 to 5 (* *p* = 0.011) compared to ginger.

The ACT score improved from randomization to day 28 of ginger (V4) (*p* = 0.019) with no further improvement at day 56 (V5) (Figure 3A). ACT did not change in the placebo group between randomization and day 28 but did improve from day 28 to day 56 (*p* = 0.012) (Figure 3B). The 2-week asthma symptom recall score improved (i.e., decrease in score) in the ginger group from the randomization visit 3 to day 28 (*p* = 0.012) and did not improve further from day 28 to day 56 (Figure 3C). There was no change in the 2-week recall score in the placebo group (Figure 3D) at either time point. The mini AQLQ score improved from randomization to day 28 in both groups, but the ginger group had a larger magnitude (mean of difference between visit 3 and 4 = −12.70, *p* < 0.001 (Figure 3E)) than the placebo group (mean of difference between randomization and day 28 = −9.08, *p* < 0.036 (Figure 3F)).

**Figure 4:**
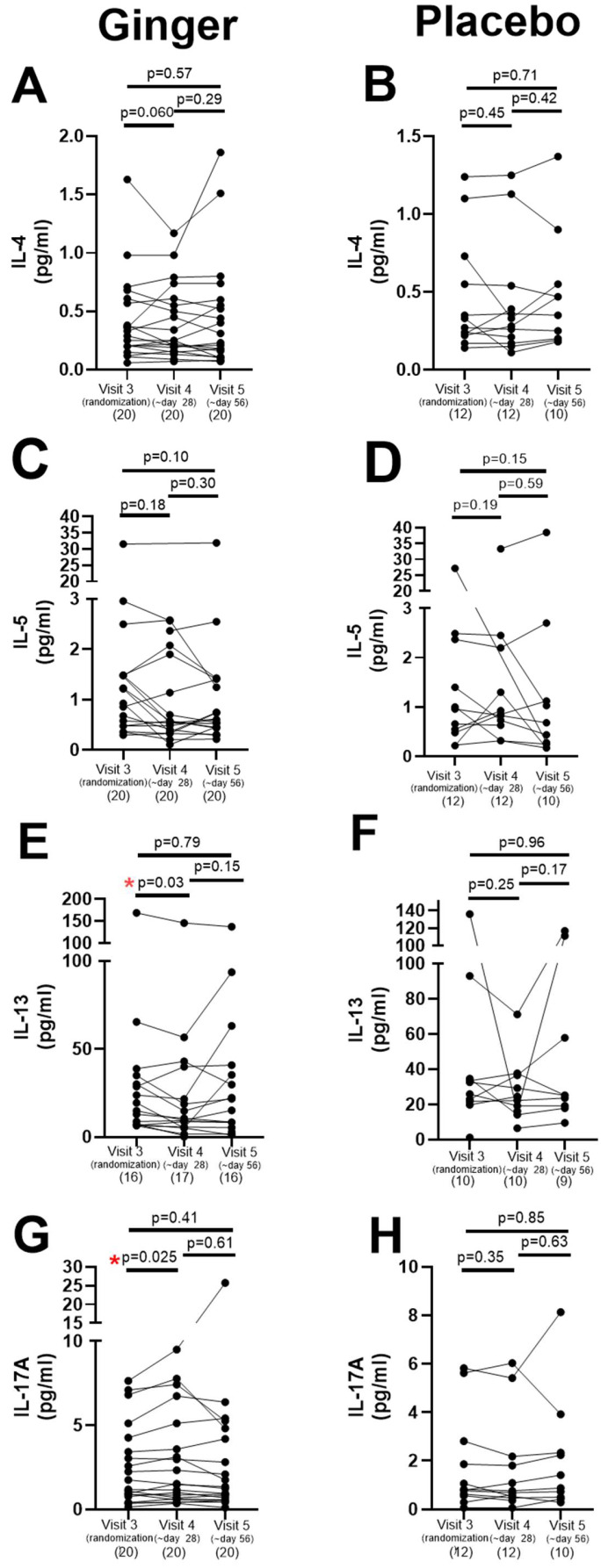
Serum cytokines (IL-4, IL-5, IL-13, and IL-17A) in study subjects at the randomization visit (visit 3) and after 28 days (visit 4) or 56 days (visit 5) of study drug. (**A**,**B**) IL-4 was not different in either the ginger or placebo group comparing visits 3 to 4 or visits 3 to 5. (**C**,**D**) IL-5 was not different in either the ginger or placebo group comparing visits 3 to 4 or visits 3 to 5. (**E**) IL-13 was significantly decreased in the ginger group (* *p* = 0.03) between visits 3 to 4 and did not decrease further between visits 4 to 5. (**F**) Placebo had no effect on IL-13 levels comparing visits 3 to 4 or visits 3 to 5. (**G**) IL-17A was significantly decreased in the ginger group between visits 3 to 4 (* *p* = 0.025) and did not decrease further between visits 4 to 5. (**H**) Placebo had no effect on IL-17A levels comparing visits 3 to 4 or visits 3 to 5.

As for serum cytokines relevant to asthma, IL-13 significantly decreased in the ginger group between the randomization visit and day 28 (n = 16, *p* < 0.03) with no further significant decrease between days 28 and 56 (Figure 4E). IL-17A significantly increased in the ginger group between the randomization visit and day 28 (n = 20, *p* < 0.025) (Figure 4G). There were no significant differences in the serum cytokine levels of IL-13 or IL-17A in the placebo group or in the levels of IL-4 or IL-5 in either the ginger or placebo groups across the 3 study visits (Figure 4). The sample size for IL-13 in the ginger group was n = 16 instead of an n = 20 at visit 3 and visit 5 and n = 17 at visit 4 because the serum levels of IL-13 were below the level of detection. In the placebo group at visit 5, the blood draw was inadequate in two subjects to measure IL-4, IL-5, IL-13, and IL-17A, yielding an n = 10 instead of 12. There were no significant changes in eotaxin, γIFN, or TNFα in either group across the three study visits.

There were no significant differences in gastrointestinal or headache side effects between ginger and placebo, nor across the three study visits. The values for the ginger group were 2.35 ± 0.41, 2.60 ± 0.40, and 1.85 ± 0.38 (mean ± SEM) (n = 20) across visits 3, 4, and 5, respectively. The values for the placebo group were 1.55 ± 0.61, 1.58 ± 0.47, and 1.50 ± 0.48 (mean ± SEM) (n = 12) across visits 3, 4, and 5, respectively. No toxicities greater than the National Cancer Institute Common Toxicity Criteria (version 2.0) grade 1 were reported in either group.

## 3. Discussion

The primary finding of this randomized, double-blinded, placebo-controlled study in mild-to-moderate asthmatics is that a twice-daily dose of 1 gm of oral ginger extract improved asthma symptoms and asthma control and modulated levels of serum cytokines related to asthma. We also confirmed, through prior studies [23,30], that this formulation and small dose of ginger extract were not associated with an increase in gastrointestinal side effects compared to placebo.

Three standard assessments of asthma symptoms and control were utilized in the present study, including the Asthma Control Test (ACT), the two-week recall asthma symptom score, and the Juniper mini Asthma Quality of Life Questionnaire (AQLQ). All three of these measurements demonstrated improvement at day 28 in the ginger group without evidence of further improvement at the 56-day time point. Interesting, there was a placebo effect in the ACT, with an improvement in asthma control between the 28th and 56th study day, and an improvement in the placebo group in the mini-AQLQ, although the ginger group had a larger magnitude of improvement when statistically comparing groups. Small placebo effects are common in asthma clinical trials, perhaps in part due to better maintenance therapy adherence by study participants [31]. There was no acute effect on FEV1 as measured one hour after the first dose of ginger ingestion and no change in FEV1 over the 56-day course of the study in the ginger or placebo group.

The initial intended primary outcomes of this study were improved tolerance for inhaled methacholine and reduction in the fraction of exhaled nitric oxide as measures of reduced lung inflammation. Our measurements of methacholine tolerance were stopped early in the study due to a forbiddance of aerosol-generating procedures during and after the COVID-19 pandemic, resulting in an insufficient number of study subjects completing this test to allow for meaningful interpretations. Exhaled nitric oxide levels did not change in response to oral ginger extract despite a change in selected asthma-associated cytokines (IL-13 and IL17-A) between visits 3 and 4. There was no change in blood eosinophilia. Perhaps the magnitude of inflammation reduction with this small daily dose of oral ginger extract was insufficient to reduce total exhaled nitric oxide or eosinophilia, or the heterogeneity of asthma endotypes in our small sample size of this pilot study did not allow for a detectable change. The direction of change in serum IL-17A (increase) in the ginger group may at first appear paradoxical because elevated IL-17A is consistently shown to be elevated in neutrophilic asthma, but whether this is pathogenic or a protective response is unclear [32]. Additionally, evidence suggests a reciprocal relationship between type 2 and type 17 airway inflammation raising the possibility that improving type 2 asthma may uncover a protective type 17 response [33]. The predominant roles of IL-17 cytokines are thought to be maintaining the epithelial cell barrier, and thus elevation may be reflective of underlying epithelial damage in asthma. Moreover, a human transcriptomics study of epithelial cells from 51 asthmatic patients revealed a negative correlation of type 2 and type 17 gene signatures, with no patient having high expression of both type 2 and type 17 gene signatures [33]. Blockade of the type 2 pathway (IL-4/IL-13) in a murine model of allergic inflammation increased Th17 expression with an increase in neutrophilic infiltration and a decrease in eosinophilic lung infiltration [33]. Despite the extensive evidence that elevated IL-17 occurs in certain endotypes of asthma (e.g., neutrophilic), multiple clinical trials of targeting a reduction in IL-17 have not been successful [34,35,36,37]. Taken together, these findings suggest a reciprocal relationship between type 2 and type 17 responses and leave open the question as to whether IL-17 is pathogenic or a reflection of a protective response.

We intentionally chose a small daily dose of ginger extract so as to not jeopardize study subject compliance with this study, as larger oral doses of daily ginger have been associated with gastrointestinal symptoms of eructation, acid reflux, or dyspepsia. We did not detect any significant gastrointestinal side effects consistent with previous findings with this same formulation of ginger extract [23,24,38]. The effects of ginger on the measured parameters may be further amplified with higher daily doses of ingested ginger.

These studies were motivated by pre-clinical studies that demonstrated that the bioactive components of ginger, gingerols and shogaols, could directly relax pre-contracted human airway smooth muscle and reduce lung inflammation and airway hyperresponsiveness in a mouse model of house dust mite antigen-induced allergic lung inflammation [21,22]. However, the pharmacokinetics of the active components of ginger are complex, undergoing conjugation as glucuronides and sulfates, and the primary component after thermal dehydration, 6-shogaol, is being metabolized extensively [39,40,41]. The bioactivity and safety of these many metabolites of the active ginger components of 6-shogaol have not been extensively studied, and therefore we chose to use a ginger extract formulation that has a proven safety record in humans that has an enriched 6-shogaol content.

Potential weaknesses of this study include an inadequate power to detect change in the initially intended primary study outcome due to COVID-19-related restrictions and methacholine responsiveness. Additionally, despite the identical appearance of the placebo and active drug, it is possible that subjects knew which arm they were randomized to based on taste or odor.

## 4. Materials and Methods

### 4.1. Clinical Asthma Study Protocol

IND approval for the administration of a dietary supplement of ginger at a dose of 1 gm twice daily was granted by the FDA (IND#126513). The study protocol was approved by the Institutional Review Board of Columbia University (protocol #AAAR8427) and by Westat^®^ acting on behalf of the National Center for Complementary and Integrative Health (NCCIH). Participants ≥ 18 years with a physician diagnosis of mild-to-moderate persistent asthma were screened for study participation in the asthma clinic of the Columbia University Medical Center. Participants taking medium-to-high-dose inhaled corticosteroids with or without a second controller therapy (long-acting beta agonist and leukotriene modifier) and poor asthma control defined as Asthma Control Test (ACT) ≤ 19 were eligible to participate. Asthma diagnosis was confirmed by an increase of either 200 mL or 12% in forced expiratory volume in one second (FEV1) after administration of 4 puffs of albuterol or a 20% reduction in FEV1 in response to inhaled methacholine up to 16 mg/mL. Patients were excluded if they had a respiratory infection within 6 weeks of screening, use of oral corticosteroids within the past 6 weeks, COPD/emphysema, current use of anticoagulation, active illness such as uncontrolled hypertension, current consumption of ginger supplements, any cigarette use in the previous 12 months, or a lifetime use of more than 10 pack years, or FEV1 < 60% predicted. Women of childbearing age were asked to use effective contraception.

### 4.2. Baseline Methacholine Challenge Testing

**Figure 5:**
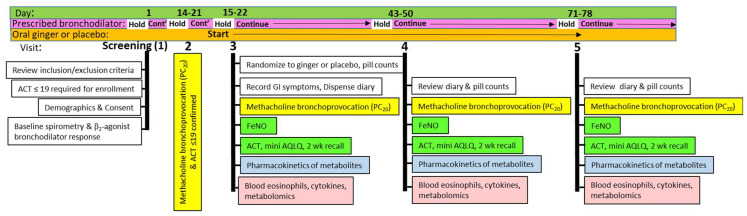
Clinical protocol timeline. Asthmatics were screened by baseline spirometry and response to β-agonist inhalation, with inadequate asthma symptom control defined as an Asthma Control Test (ACT) of 19 or less. Fractional exhaled nitric oxide (FeNO), methacholine bronchoprovocation, blood samples, and symptom scores were measured over 56 days.

Following a screening visit (visit 1), a 14–21 day run-in period occurred during which patients were maintained on their usual asthma therapies. Patients who remained exacerbation--free and continued to have ACT ≤ 19 and FEV1 > 60% predicted underwent baseline methacholine bronchoprovocation testing (visit 2) 1–2 days prior to the randomization visit (visit 3) (Figure 5).

For those subjects who underwent methacholine provocation to determine study eligibility, the test was not repeated. Randomization codes and allocations were generated by an independent statistician and provided to the research team through RedCap. RedCap is an institutionally available automated system for randomization. All study team members were blinded to the randomization code.

At each study visit starting at randomization, short-acting bronchodilators were held for 6 h, and long-acting bronchodilators were held for 36 h prior to the visit. Basic spirometry, fractional exhaled nitric oxide (FeNO) (Circassia Niox^®^ Vero, Morrisville, NC, USA), the Asthma Control Test (ACT) score, the Juniper mini Asthma Quality of Life Questionnaire (AQLQ) score, and a 2-week asthma symptom recall were obtained. Methacholine hyperresponsiveness at day 28 and day 56 was conducted only if patients met safety criteria (FEV1 > 1.5 L and > 60% predicted).

### 4.3. Randomization

At the randomization visit, patients were assigned in a 1:1 ratio to receive ginger 1 gm twice per day (Pure Encapsulations^®^) or a matching placebo for a period of 56 days. Both the ginger capsules and identically appearing placebo capsules were a kind gift from Pure Encapsulations^®^. Spirometry was measured at the randomization visit one hour following ingestion of the study drug for assessment of immediate bronchodilatory properties.

Blood samples were obtained at visits 3, 4, and 5 for measurement of eosinophil count and multiplex analysis of selected human cytokines (Eve Technologies, Calgary, AB, Canada). Pill count was recorded at each visit. A questionnaire was included with the ACT, 2-week recall, and AQLQ assessments that queries possible adverse events associated with oral ginger using the NCI and NIH Criteria for Adverse Events (CTCAE) Version 4.0. The criteria included the absence or presence of meteorism, flatus, eructation, flatulence, nausea, vomiting, diarrhea, or headache. The total number of symptoms were compared between the ginger and placebo group and across visits 3, 4, and 5.

### 4.4. Pharmacokinetic Study

8 participants underwent pharmacokinetic measurements of the key metabolites of active ginger components over 7 time points (pre-dose, 0.5 h, 1 h, 2 h, 3 h, 4 h, and 8 h) at randomization, day 28, and day 56 (i.e., visits 3, 4, 5). Results of these measurements have been previously published [42].

The primary endpoints were a change in the methacholine PD20 (dose of methacholine that causes a 20% decline in FEV1) or a reduction in FeNO. Secondary endpoints included FEV1, ACT, Juniper mini AQLQ, and a 2-week asthma symptom recall. Additional secondary outcomes included a decrease in blood eosinophilia or selected cytokines (IL-4, IL-5, IL-13, eotaxin, IFNγ, TNFα, and IL-17A). Exploratory analyses included a measurement of an acute (one hour following study drug ingestion at V3) improvement in spirometry.

Due to an institutional research pause caused by the COVID-19 pandemic, 8 subjects were dropped from the study during a COVID-19 pause. Upon resumption of study activities in May 2020, study procedures were modified due to an institutional hold on aerosol-generating procedures, which led to the discontinuation of methacholine bronchoprovocation in study participants.

### 4.5. Statistical Design and Power

We estimated a 5% improvement in inhaled methacholine tolerance as clinically significant comparing before and after treatment with the study drug, with a standard deviation (SD) of 7.5%. Assuming a 10% drop-out rate, we planned to enroll 18 subjects per treatment arm to achieve 80% power at the 0.05 significance level.

### 4.6. Statistical Methods

We examined if there are short-term treatment effects (1 h after study drug) on spirometry measures within the ginger group and within the placebo group separately using a paired *t*-test. We then examined if short-term effects are different or not between the ginger and placebo groups using two-sample *t*-tests by comparing changes in measures from baseline to 1 h after ginger or placebo administration. We then compared chronic long-term effects of ginger or placebo on spirometry, FeNO, measurements of asthma symptoms (ACT, 2-week recall, and AQLQ), serum cytokines, and blood eosinophils. Similarly, for long-term effects, we compared changes in measures from randomization (visit 3) to visit 4 and visit 5, or between visits 4 and 5, within ginger and placebo groups using paired *t*-tests. In addition, we examined whether the overall long-term effects of the ginger group and placebo group are different or not using a linear mixed model (LMM) with a random intercept, where repeated outcomes are changes in measures from baseline to that of visit 4 (day 28) and visit 5 (day 56), and the main predictor of interest is the indicator of being in the ginger or placebo groups.

## 5. Conclusions

We demonstrated for the first time that a relatively low dose of 1 gm twice daily of oral ginger extract improved asthma symptoms and asthma control in uncontrolled mild-to-moderate asthma over 28 days and modulated the serum levels of asthma-associated cytokines without gastrointestinal side effects. This is the first human-controlled study to demonstrate that the oral ingestion of a dietary supplement of ginger can improve asthma symptom control. It remains to be determined if higher daily doses of oral ginger extract can further improve asthma symptom control by objective and subjective measures and whether specific endotypes of asthmatics selectively demonstrate benefit.

## Figures and Tables

**Table 1:** Baseline values of asthmatic subjects before randomization, visit 2. ACT: Asthma Control Test; AQLQ: Asthma Quality of Life Questionnaire; BMI: body mass index; FeNO: fractional exhaled nitric oxide; FEV1: forced expiratory volume in one second; FVC: forced vital capacity; IL: interleukin; PD_20_: provocation dose causing a 20% decline in FEV1.

	Ginger (n = 20)	Placebo (n = 12)	*p*-Value *t*-Test
Age (years)	53.9 ± 17.5	56.1 ± 13.0	0.71
Sex (female n, %)	11, 55	7, 58	
BMI (kg/m^2^)	29.4 ± 5.5	30.6 ±6.4	0.60
Hispanic Ethnicity (%)	55	42	
Race (Black, White, Asian, more than one race, %)	20, 45, 15, 20	33, 25, 9, 33	
Pre-albuterol FEV1 (L) (% predicted)	2.13 ± 0.82, 73.8 ± 18.8	2.19 ± 0.52, 75.2 ± 19.5	0.65, 0.60
Post-albuterol FEV1 (L) (% predicted)	2.12 ± 0.85, 71.3 ± 16.7	2.06 ± 0.51, 71.5 ± 20.8	0.99, 0.71
Pre-albuterol FEV1/FVC	0.71 ± 0.10	0.74 ± 0.11	0.27
Post-albuterol FEV1/FVC	0.71 ± 0.92	0.72± 0.09	0.49
PD_20_ methacholine	9.24 ± 12.09	9.44 ± 15.0	0.94
FeNO (ppb)	30.5 ± 28.78	72.7 ± 83.8	0.20
ACT	14.2 ± 3.23	15.8 ± 2.52	0.13
Mini AQLQ	65.1 ± 16.0	58.1 ± 17.6	0.35
Two-week symptom score	6.55 ± 2.48	5.17 ± 3.10	0.17
Eosinophil count (×10^9^/L)	0.24 ± 0.23	0.30 ± 0.28	0.57
Eosinophil % of total WBC	3.8 ± 3.3	4.1 ± 3.6	0.83
IL-4 (pg/mL)	0.42 ± 0.37	0.46 ± 0.37	0.75
IL-5 (pg/mL)	3.56 ± 7.58	8.82 ± 20.2	0.30
IL-13 (pg/mL)	30.0 ± 40.3	42.1 ± 40.6	0.47

**Table 2:** Short-term effects comparing spirometry measures at baseline and 1 h after study drug within the ginger group and within the placebo group (mean ± S.E.). No significant short-term effects were found within either group using a paired *t*-test. FEV1: forced expiratory volume in one second; FVC: forced vital capacity; L: liter.

Measure	Group	N	Baseline	1 h After Drug	Paired *t*-Test *p*-Value
FEV1%	Ginger	20	73.75 ± 4.2	71.33 ± 3.94	0.58
FEV1%	Placebo	12	75.17 ± 5.62	71.45 ± 6.28	0.52
FVC%	Ginger	20	81.35 ± 2.8	80.37 ± 3.34	0.37
FVC%	Placebo	12	80.58 ± 4.63	77.27 ± 5.19	0.26
FEV1/FVC	Ginger	20	0.71 ± 0.02	0.71 ± 0.02	0.43
FEV1/FVC	Placebo	12	0.74 ± 0.03	0.72 ± 0.03	0.76
FEV1 (L)	Ginger	20	2.12 ± 0.18	2.12 ± 0.2	0.49
FEV1 (L)	Placebo	12	2.18 ± 0.15	2.06 ± 0.15	0.46
FVC (L)	Ginger	20	2.98 ± 0.21	2.95 ± 0.21	0.14
FVC (L)	Placebo	12	3.02 ± 0.2	2.9 ± 0.22	0.14

**Table 3:** Chronic treatment effects are estimated from a linear mixed-effects model with a random intercept adjusting for baseline measures. No significant treatment effect was found on any of the five spirometry measures. CI: confidence interval; FEV1: forced expiratory volume in one second; FVC: forced vital capacity.

Measure	Term	Estimate (95% CI)	*p*-Value
FEV1%	Ginger Group	−0.71 (−7.95, 6.52)	0.82
FEV1 (L)	Ginger Group	−0.02 (−0.22, 0.18)	0.86
FVC (L)	Ginger Group	−0.04 (−0.24, 0.15)	0.65
FVC%	Ginger Group	−0.38 (−6.16, 5.4)	0.89
FEV1/FVC	Ginger Group	0.01 (−0.02, 0.04)	0.61

## Data Availability

Deposited at clinicaltrials.gov Registration: NCT03705832, 10 October 2018.

## Abbreviations

ACT	Asthma Control Test
AQLQ	Asthma Quality of Life Questionnaire
ASHMI	Anti-Asthma Herbal Medicine Intervention
ASM	airway smooth muscle
CAM	complementary and alternative medicine
CTCAE	common terminology criteria for adverse events
FDA	Food and Drug Administration
FeNO	fractional exhaled nitric oxide
FEV1	forced expiratory volume in one second
FVC	forced vital capacity
IFNγ	interferon gamma
IL	interleukin
MLC	myosin light chain
NCCIH	National Center for Complementary and Integrated Health
NCI	National Cancer Institute
NIH	National Institutes of Health
PD_20_	dose of methacholine that causes a 20% fall in FEV1
TNFα	tumor necrosis factor alpha
V3	visit 3
V4	visit 4
V5	visit 5
